# Necdin, a p53 target gene, in stem cells

**DOI:** 10.18632/oncotarget.997

**Published:** 2013-04-30

**Authors:** Takashi Asai, Yan Liu, Stephen D Nimer

**Affiliations:** Molecular Pharmacology and Chemistry Program, Sloan-Kettering Institute, Memorial Sloan-Kettering Cancer Center, New York, New York and Sylvester Comprehensive Cancer Center, Miller School of Medicine, University of Miami, Miami, FL; Department of Pediatrics, Herman B Wells Center for Pediatric Research, Indiana University School of Medicine, Indianapolis, IN; Molecular Pharmacology and Chemistry Program, Sloan-Kettering Institute, Memorial Sloan-Kettering Cancer Center, New York, New York and Sylvester Comprehensive Cancer Center, Miller School of Medicine, University of Miami, Miami, FL

Necdin, a member of the melanoma antigen gene (MAGE) family, originally identified to suppress cell proliferation in post-mitotic neurons, facilitates the entry of the cell into *cell cycle, functions like retinoblastoma (Rb) protein, and acts as a transcriptional repressor that recognizes* guanosine (G)-rich DNA sequences. Necdin also binds to p53 and inhibits p53-dependent apoptosis in these cells, demonstrating its function as an endogenous anti-mitotic and anti-apoptotic protein in post-mitotic neurons [1]. The *necdin* gene is located on chromosome 15 in human and its expression is controlled through genomic imprinting; it is one of the genes that have been associated with the human Prader-Willi syndrome, a genetic neuro-behavioral disorder [2]. Necdin is expressed in some stem or undifferentiated progenitor cells such as mesoangioblast stem cells [3], vessel-associated stem cells [4], adipocyte progenitor cells [5, 6], and hematopoietic stem cells (HSCs) [7], yet the function of necdin in these cells remains unclear.

While highly expressed in long-term HSCs, transcript profiling of hematopoietic stem/progenitor cells (HSPCs) isolated from wild-type and p53 null mice identified *necdin* as being a p53 target gene [7]. We then demonstrated using chromatin immunoprecipitation assays that the *necdin* promoter contains a p53 binding site and that necdin is a direct transcriptional activator of p53 in HSPCs. Lentivirus-mediated knock down or over-expression of necdin resulted in decreased or increased HSPC quiescence, respectively [7].

We recently clarified the function of necdin in HSCs and HSPCs using necdin null mice [8]. Necdin null mice show the same phenotype as the patients with Prader-Willi syndrome and because they die perinatally, we transplanted necdin null fetal liver cells into lethally irradiated wild type recipients to reconstitute their bone marrow function and analyzed the HSC intrinsic function of necdin in adult hematopoiesis. Necdin-null HSCs have normal self-renewal capacity after serial transplantation, while we found that necdin-null HSCs are less quiescent and more proliferative than normal, suggesting that necdin has the similar function as p53 in promoting HSC quiescence in the steady state. We then demonstrated that necdin-null HSCs are highly sensitive to genotoxic stress, irradiation or chemotherapy, with increased apoptosis via both cell-cycle-dependent and cell-cycle independent mechanisms. We further found that the function of necdin in hematopoiesis in response to genotoxic stress depends on Gas2L3, a novel member of the Gas2 family, using lentivirus-mediated knock down or over-expression of Gas2L3 in HSPCs [8]. Therefore, necdin appears to function as a molecular switch in adult hematopoiesis: to promote HSC quiescence in a p53-like manner in the steady state, but to suppress p53-dependent apoptosis under the stress conditions.

Acute leukemia is thought to begin in rare leukemia-initiating cells (LICs) that maintain or re-acquire the capacity for indefinite self-renewal through accumulated mutations and/or epigenetic changes. LICs, particularly those in a quiescent state, are thought to be resistant to chemotherapy or targeted therapies, and it is hypothesized that leukemia relapse may occur because current therapies just eliminate proliferating cells, but fail to eradicate quiescent LICs that can reinitiate the disease once a patient is in remission. Therefore, the development of new therapeutic approaches that can target LICs may have an impact on our ability to eradicate acute leukemia. Considering that necdin-null HSCs are more proliferative and show enhanced sensitivity to chemotherapy, or irradiation, targeting necdin may provide a possible therapeutic approach to eliminating quiescent LICs.

The functions of necdin in other stem or progenitor cells have recently been reported. Necdin is highly expressed in adipocyte progenitor cells and knockdown of necdin accelerates adipocyte differentiation and proliferation [5]. Similarly, necdin null mice show enhanced adipocyte progenitor cell proliferation and increased white adipocyte expansion [6]. These functions of necdin in adipocyte progenitor cells are similar to those seen in HSPCs. Necdin is also significantly expressed in vessel-associated stem cells, such as mesoangioblasts (MABs); in this system, the overexpression of necdin in MABs enhances myogenic differentiation and improves cell survival after the toxin administration [4]. Thus, the functions of necdin in stem or progenitor cells vary depending on the cell context.

Further studies are needed to clarify the function of necdin in various normal and cancer stem cell populations. However, based on enhanced chemosensitivity, and radiosensitivity, identified in necdin null HSCs, necdin may be a promising therapeutic target to enhance the killing of dormant, cancer stem cells.
